# The significant costs of prostate cancer management—An analysis from a Caribbean hospital

**DOI:** 10.1002/bco2.70003

**Published:** 2025-04-02

**Authors:** Geneva Pantoja, Amanda Wibben, Visham Bhagaloo, Sara Seetaram, Satyendra Persaud

**Affiliations:** ^1^ Baylor College of Medicine Houston Texas USA; ^2^ Georgetown University School of Medicine Washington DC USA; ^3^ San Fernando Hospital San Fernando Trinidad and Tobago; ^4^ University of the West Indies St. Augustine Trinidad and Tobago

**Keywords:** cost‐of‐illness, economic impact, prostate cancer, Trinidad

## Abstract

**Objectives:**

The objective of this paper is to analyse the annual costs associated with the diagnosis and treatment of prostate cancer at San Fernando General Hospital, a large, public hospital in Trinidad.

**Materials and Methods:**

A prospective cost‐of‐illness study analysed 1 month of all prostate cancer‐related events at San Fernando General Hospital. These included inpatient admissions, outpatient visits and surgical procedures. All resources utilized during these visits were logged, itemized and assigned a cost via the Costing Unit of San Fernando General Hospital (SFGH). The annual cost was extrapolated.

**Results:**

Two hundred eight outpatient visits and eight acute presentations were recorded for the month in addition to scheduled surgical presentations. An estimated 2496 outpatient visits and 96 acute admissions occurred annually. The mean age of presenting patients was 69 years old, with the majority (61%) of patients of African ethnicity. The overall estimated cost to the Regional Health Authority was TTD $14052157.66 (USD $2066493). Outpatient visits related to screening, diagnosis or management of prostate cancer, including prostate biopsies, comprised the majority of the costs (57%), while nephrostomies related to upper urinary tract obstruction from prostate cancer contributed least (0.7%). The total cost of metastatic disease was disproportionate to its presentation, with 17% of cases being metastatic but contributing 32% toward overall cost. Of cases with a documented chief complaint, visits related to cancer diagnostics, presentations with symptoms of prostate cancer and biopsies contributed to two thirds of the total cost, while follow‐up visits for previously diagnosed prostate cancer contributed to one third.

**Conclusion:**

This analysis shows that prostate cancer has a significant financial burden on the Trinidadian economy. Efforts must be dedicated to early screening and prevention, and policymakers should be aware of the economic impact of the disease when making budgetary allocations. Although diagnostics can be costly, it likely minimizes larger costs associated with extensive treatment and acute hospitalizations related to metastatic disease, as seen in this study.

## 1 INTRODUCTION

In 2020, nearly 1.4 million new cases of prostate cancer were diagnosed, accounting for 7.3% of all cancer diagnoses globally. On the twin islands of Trinidad and Tobago specifically, prostate cancer is the most prevalent cancer overall as well as the most common cause of cancer‐related mortality.^1^ Despite the country's status as a high‐income country and its access to some first line treatments, its incidence and mortality rates from prostate cancer are among the highest in the world.^2^ This is in contrast to the rest of the world, where prostate cancer ranks third in incidence and eighth in mortality across all genders globally.^3^ Although the exact cause of prostate cancer is unknown, genetic and environmental factors are thought to play a large role. Various aspects, such as the role of Human Herpes Virus 8 and the ELAC and RANSEL genes were identified in the Tobago Prostate Cancer Screening Study, which are thought to contribute to the disease's high prevalence in the country.^4^

Despite its prevalence, there is little literature regarding the cost of diagnosis and treatment of prostate cancer in Trinidad and Tobago. Healthcare in Trinidad and Tobago exists as a two‐tiered system, with both public and private hospitals. Because care at public hospitals is provided at no cost, patients in Trinidad and Tobago widely utilize public infrastructure. The main public hospitals on the islands are the Port of Spain General Hospital (POSGH), San Fernando General Hospital (SFGH), Eric Williams Medical Sciences Complex (EWMSC), Sangre Grande Hospital, Arima General, Point Fortin Hospital and Scarborough Hospital in Tobago. SFGH is located in South Trinidad and houses the country's largest urological unit with a 24‐bed ward, caring for a large portion of the country's diverse population of 1.3 million.

Annual visits consisting of a digital rectal exam (DRE) and a serum prostate‐specific antigen (PSA) test are the standard of care for men 45 and older, although Trinidad and Tobago lack specific national screening guidelines. While PSA testing is widely available at private laboratory facilities, many patients choose to utilize labs in the public hospital system. If a DRE or PSA is found to be abnormal, a transrectal ultrasound‐guided biopsy is recommended. These biopsy services are offered at SFGH, EWMSC, Arima General, POSGH, Sangre Grande and Scarborough, in addition to several private hospitals. Depending on the results of the DRE, PSA, biopsy and Gleason score, certain imaging modalities may be recommended to guide treatment options. Imaging options include X‐ray, CT and MRI to classify the extent of the disease. Once diagnosed, various first line options are available for the treatment of prostate cancer in Trinidad and Tobago, including active surveillance, radical prostatectomy, interstitial prostate radiation/brachytherapy, androgen deprivation and external beam radiation, treatments that remain inaccessible to many middle‐ and low‐income countries.^5^ These treatments also account for a substantial percentage of urological services provided across the island.

Still, prostate cancer incidence and mortality remains near the highest in the world despite the country's growing access to advanced treatment methods.^2^ This suggests that other unmet needs and disparities likely remain to account for the country's high cancer burden. Some studies have pointed to ethnic disparities particularly among patients of African descent. This includes allelic differences and diagnostic factors like higher PSA levels and earlier age at time of diagnosis.^6^ However, much remains unknown about the relationship between individual factors, like family and exposure history, and genomic discrepancies that could explain the high morbidity and mortality among Afro‐Caribbean men in Trinidad and Tobago in particular.^7^ Additionally, some studies point to cultural and institutional barriers that delay prostate cancer care in Trinidad.^8^

The high incidence and prevalence of prostate cancer remains a pressing issue for Trinidad and Tobago as well as for other similar Afro‐Caribbean nations like Barbados and Cuba who face similar morbidity and mortality rates.^2^ Given Trinidad and Tobago's resources as a high‐income country with growing access to advanced prostate cancer diagnosis and treatment methods, the country is well‐positioned to improve on its high prostate cancer rates. However, this will require a deep understanding of the country's expenditure on prostate cancer care and screening in order to guide resource allocation. To the best of our knowledge, there have been no studies thus far examining the cost of these medical services in Trinidad and Tobago. Given the high prevalence of the disease and the limited resources of Trinidad's public healthcare system, it is crucial to determine the medical costs associated with prostate cancer and to elucidate which services and patient populations contribute most significantly to these costs. This cost‐of‐illness study will investigate the approximate yearly economic impact of prostate cancer on SFGH specifically, guiding policymakers and other important stakeholders in the healthcare system to allocate resources accordingly with the goal of impacting the internationally high incidence and prevalence of prostate cancer. Additional information on the different populations affected by prostate cancer will also shed light on disparities in medical care costs and healthcare utilization.

## 2 PATIENTS AND METHODS

This was a cross‐sectional cost‐of‐illness study conducted over 5 weeks at the San Fernando Hospital in Trinidad and Tobago. Patients matched inclusion criteria if they were newly diagnosed with prostate cancer, following up for a previous diagnosis, receiving treatment or presenting for an appointment to investigate suspicion of prostate cancer.

Data were collected daily via paper survey of each patient presenting for prostate cancer‐related care to any SFGH department. Patients were surveyed during appointments in the outpatient clinic or upon presenting for biopsy or treatment (i.e., surgery, goserelin injections, chemotherapy and radiation). Patients housed in the urology unit for acute complications or inpatient admittance were also surveyed daily. Additional demographic data were also collected by reviewing patients' paper charts to collect patient age, ethnicity, town of residence, chief complaint, Eastern Cooperative Oncology Group (ECOG) performance status and disease stage.

The direct medical costs of each visit, diagnostic procedure or intervention, including surgery, were obtained from the Costing Department of the SFGH and the Trinidad and Tobago Ministry of Health. This included compensation of medical staff, labs, imaging, prescription medications and any other interventions involved in the treatment of symptoms or management of prostate cancer care provided in the urology ward and outpatient clinic at SFGH. The sum of these costs was taken to yield an estimate of the total monthly cost. These data were then extrapolated to estimate the total cost for the year. To obtain the most accurate data on surgical procedures, goserelin injections and radiotherapy administration related to prostate cancer treatment, records from the past year (June 2022–June 2023) were obtained. This was done to account for discrepancies in prostate cancer‐related care for these particular treatments, which varied monthly due to fluctuations in functional equipment and attending availability. The sum of the extrapolated data from the surveys in addition to the annual data from surgical procedures and goserelin injections were taken to estimate a total yearly cost for the diagnosis and treatment of prostate cancer at SFGH.

## 3 RESULTS

### 3.1 Demographic information

A total of 250 patients presented between 11 June 2023 and 31 July 2023, the study's 5‐week survey period. The reported ages ranged from 40 to 93 years, with a mean of 69.24 years. Nearly 90% of the patients seen for prostate cancer care or investigation were patients aged ≥60 years. Of the 250 cases, there were 247 recorded locations of residence. Of the 247 recorded, 206 patients (83.40%) resided in South Trinidad, 31 (12.55%) in Central Trinidad, seven (2.83%) in East Trinidad and three (1.21%) in North Trinidad. Patient ethnicities were distributed as follows: 153 (61.2%) African, 71 (28.4%) East Indian, 25 (10.0%) African‐East Indian mixed and 1 (0.4%) other. Metastatic disease was present in 20% and 14% of African patients and East Indian patients, respectively, surveyed over the 5‐week period. One hundred seventy‐four patients (59% over 5 weeks) presented with a baseline ECOG status of 0; 24 patients (9.7%) with a status of one; 28 patients (11.3%) with a status of two; 12 patients (4.8%) with a status of three; and 10 patients (4%) with a status of four. Two cases did not have an ECOG status documented. These demographic data are depicted in Table 1.

**TABLE 1:** Demographic information and presentations.

**Age (mean)**	69.24
**Location of Residence**	**# of Patients (during study period)**
South Trinidad	206 (83.40%)
Central Trinidad	31 (12.55%)
East Trinidad	7 (2.83%)
North Trinidad	3 (1.21%)
**Ethnicity**	
African	153 (61.2%)
East Indian	71 (28.4%)
Mixed	25 (10.0%)
Other	1 (0.40%)
**Metastatic disease**	
Metastatic	42 (67.2%)
Non‐metastatic	195 (78.0%)
Undetermined	13 (5.2%)
**ECOG status**	
0	174 (59.0%)
1	24 (9.7%)
2	28 (11.3%)
3	12 (4.8%)
4	10 (4.0%)
**Care required at presentation**	**Estimated annual #**
Diagnostics	1872 (68.2%)
Treatment of PC	752 (27.4%)
Disease complication management	120 (4.4%)

*Note*: This table provides specific demographic information, Eastern Cooperative Oncology Group Performance Status (ECOG) and stage of care at presentation for the 250 case presentations recorded during the study period. Annual estimates are listed for care required at presentation where prostate biopsies and urology outpatient visits are considered diagnostics; goserelin, bilateral scrotal orchiectomy (BSO), radical prostatectomy and radiotherapy are considered treatment; acute management, nephrostomy and other emergent procedures are considered disease complication management.

### 3.2 Patient visits and costs

In the 5‐week survey window, 184 patients were seen in the urology outpatient clinic; 24 patients were seen in the oncology outpatient clinic; 34 patients were seen for elective prostate biopsies; and eight patients were admitted to the urology ward for prostate cancer‐related care. Of the eight patients presenting acutely in SFGH, four received bilateral scrotal orchiectomies as treatment, and two received inpatient biopsies for high suspicion of prostate cancer. One death was reported. These numbers were then extrapolated to estimate the number of cases seen over 1 year at SFGH, as detailed in Table 2.

**TABLE 2:** Direct annual extrapolated medical costs by presentation.

Service provided	Estimated annual #	Cost (TTD)	% of Total cost
**Urology outpatient visits**	2208	4818298.32	34.3
**Goserelin**	636	1443141.24	10.3
**Biopsies**	408	2670888.24	19.0
**Oncology outpatient visits**	288	461676.48	3.3
**Acute**	96	1921749.36	13.7
**Bilateral scrotal orchiectomies**	54	124279.38	0.9
**Radiotherapy**	40	2060800.00	14.7
**Nephrostomies (insertion/exchange/removal)**	24	98361.36	0.7
**Radical prostatectomies**	22	452963.28	3.2
**Total**	**3776**	**14052157.66**	**100**

*Note*: Individual costs for each service were provided by San Fernando General Hospital (SFGH) and the Trinidad and Tobago Ministry of Health. These costs were multiplied by the surveyed quantity of each service and extrapolated annually to provide the annual cost of each service. The percentage of total costs of prostate cancer‐related services is visually represented in Figure 1

**FIGURE 1:**
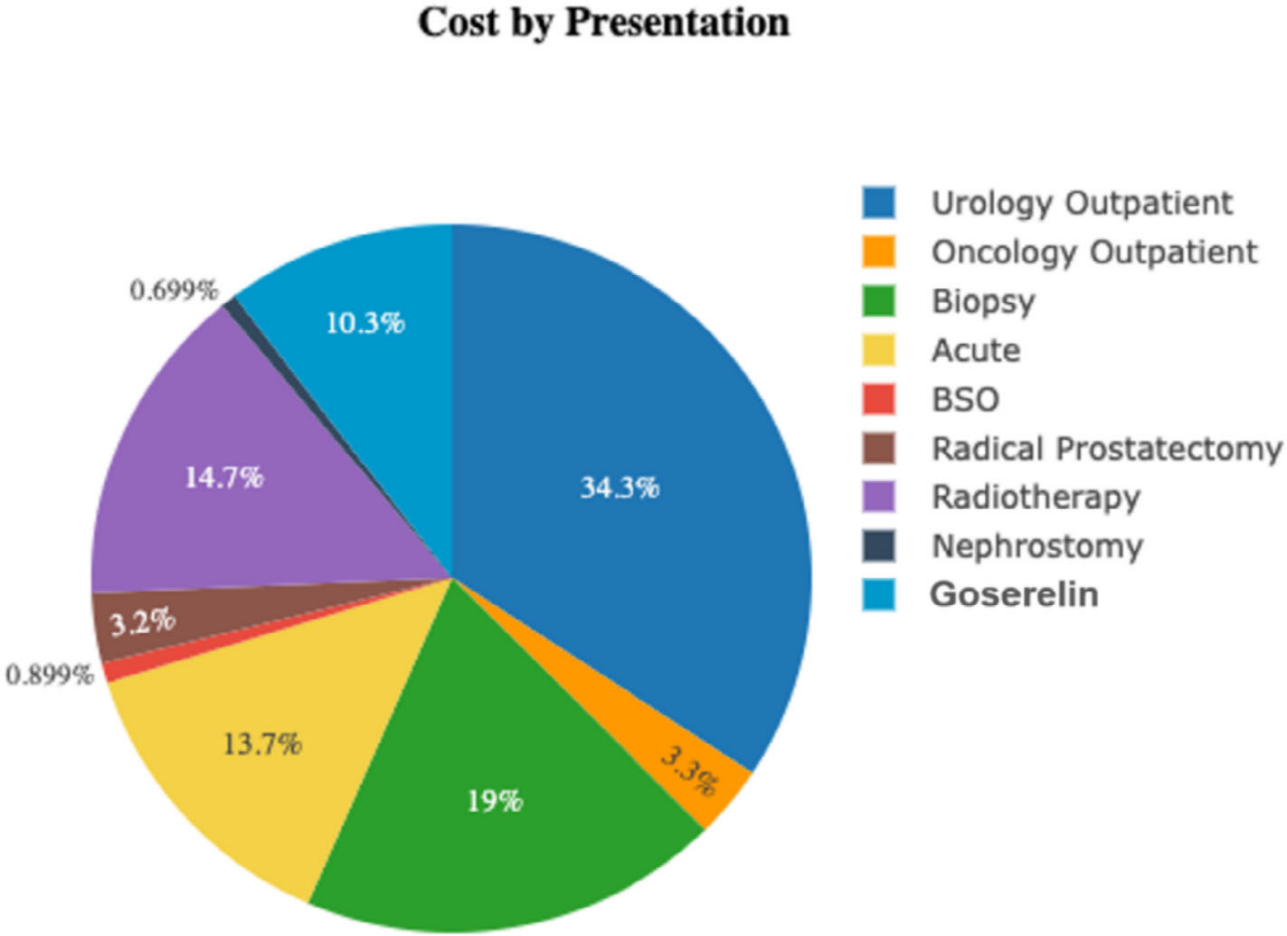
Percentage of total cost by presentation: Costs were estimated based on the data extrapolation shown in Table 1 and then presented here as a percentage of the total cost of prostate cancer‐related care, estimated to be TTD $14052157.66 annually (USD $2071021.05).

Over the past year, 54 patients underwent bilateral scrotal orchiectomy, 636 underwent goserelin injections, 40 underwent radiotherapy treatment, 24 underwent nephrostomy insertion exchange or removal related to complications of their prostate cancer and 22 underwent radical prostatectomy, as shown in Figure 1. The estimated 1‐month cost associated with collected patient presentations was TTD $1171013.13 (USD $172554.41; 1 TTD = 0.15 USD). The total extrapolated direct medical cost of prostate cancer was then found to be TTD $14052157.66 annually, which equates to USD $2071021.05. Of the total direct medical costs, costs related to outpatient urology visits and elective investigative visits (biopsies) represent more than half (53.3%) of the total cost.

For scheduled cases seen in urology and oncology outpatient clinics, acute presentations in the urology ward, and elective appointments for biopsies, the cost distribution by metastatic disease versus non‐metastatic disease was also analysed. Of the 250 cases surveyed over the 5‐week period, 42 cases were identified with metastatic disease, 195 lacked diagnoses of metastatic disease at the time surveyed, and 13 were undetermined. Despite metastatic cases representing just 16.8% of the total presentations in the 5‐week period, the cost of metastatic disease accounted for 31.8% of the total cost of prostate cancer‐related care at SFGH, nearly double in cost relative to the prevalence of cases.

The mean cost for a case with metastatic disease was calculated to be TTD $6187.58, while the mean cost for a case of non‐metastatic disease was only TTD $2678.39. Of those who were seen in clinic patient, visits for prostate cancer follow‐up were responsible for 33.4% of costs. These costs are outlined in Table 3.

**TABLE 3:** Type of service received for metastatic and non‐metastatic disease.

Type of service	Non‐metastatic		Metastatic		Undetermined	
	Costs (TTD)	Incidence (%)	Costs (TTD)	Incidence (%)	Costs (TTD)	Incidence (%)
Outpatient visit urology/oncology visit	310124.97	77.9	93636.63	15.9	32043.95	6.3
Biopsy	209, 481.72	94.1	6546.15	2.9	6546.15	2.9
Acute	0	0	159659.78	100	0	0

Table 3 details the cost and incidence of metastatic versus non‐metastatic disease out of outpatient visits, elective biopsies, and acute presentations across the 5‐week survey period.

Costs stratified by ethnicity are shown in Table 4. The overall cost for all African patients seen in outpatient clinics, for biopsies and acutely in the urology ward was found to be TTD $549286.84; TTD $186 117 for East Indian patients; and TTD $73070.14 for mixed ethnicity patients. The average cost per case was TTD $3590.11 for a patient of African descent, TTD $2621.37 for a patient of East Indian descent and TTD $2992.81 for a patient of mixed descent.

**TABLE 4:** Total and average cost per case by ethnicity.

Ethnicity	% of cases	Outpatient, biopsy and acute total cost (TTD)	Avg. cost per case (TTD)
**African**	61.2	549286.84	3590.11
**East Indian**	28.4	186117.00	2621.37
**Mixed**	10.0	73070.14	2992.81
**Other**	0.4	9601.07	9601.07

*Note*: Table 4 indicates the cost per case of prostate cancer‐related care as a function of patient ethnicity across the 5‐week survey period.

## 4 DISCUSSION

This comprehensive cost‐of‐illness study was primarily conducted to estimate the approximate yearly economic impact of prostate cancer at SFGH specifically. Secondary data, such as patient demographics, were also collected to better understand the makeup of prostate cancer patients presenting to SFGH. The findings of this study, along with the high prevalence of prostate cancer in Trinidad and Tobago, largely indicate that prostate cancer diagnosis and treatment places a considerable financial burden on the country's healthcare system.^3^ Prostate cancer accounts for a large proportion of urological burden at SFGH with the other major urological illness being urolithiasis. The self‐reported incidence of kidney stones in Trinidad and Tobago is over 16%; yet total annual expenditure on kidney stones at SFGH is estimated at just under TTD $12 million (unpublished data), which is less than expenditures for prostate cancer.

The largest medical costs were associated with outpatient urology visits, elective procedures such as prostate biopsies and radiotherapy treatment. Outpatient visits and biopsies were by far the costliest expenses, and these accounted for approximately 76.9% of the total yearly cases, either for diagnostic purposes or for follow‐up of previously diagnosed prostate cancer. Out of all the treatments surveyed, radiotherapy was the most costly, both in total and per patient. This finding is in line with other prostate cancer cost‐of‐illness studies that reveal radiotherapy as one of the highest cost drivers.^9^ Because non‐metastatic prostate cancer has a relatively good prognosis, with a 5‐year survival rate of approximately 100% compared to 30% for metastatic prostate cancer, early diagnosis and treatment of localized prostate cancer is essential.^10^

Although the majority (78%) of cases presented with non‐metastatic disease, these cases accounted for a disproportionately lower percentage of the total costs of prostate cancer care. The mean cost for cases for patients suffering from metastatic disease was 2.3 times the mean cost for cases for patients with non‐metastatic disease. This highlights the disproportionate cost of metastatic disease, likely because these patients often present acutely with symptoms related to their disease and require much more invasive care than those who present for diagnostics or for standard non‐metastatic disease treatment. This finding corroborates other studies showing that direct costs increase with the progression of the disease.^9^ Taken together, these findings point to the burden of treating metastatic disease and make a striking case for directing resources toward early detection. Therefore, continuing to invest in outpatient screening and diagnosis for prostate cancer and diagnosing patients at an earlier stage of disease is likely to reduce overall costs associated with additional rounds of treatment or acute hospital stays related to prostate cancer complications. Additional studies analysing the cost of prostate cancer at each specific stage, for example, castrate resistant versus non‐resistant metastatic disease, would also help elucidate more specifically which patient groups bear the highest costs.

Furthermore, these results demonstrate that the cost of an orchidectomy is significantly less than administration of ADT. This is even further magnified considering that ADT injections are administered on a quarterly basis as compared to a single surgery for an orchiectomy. Significant cost savings could be achieved by utilizing orchiectomy whenever possible.

The mean age of the patients surveyed was 69 years, and nearly 90% of the cases were over 60 years old, consistent with the higher prostate cancer burden among older men that is well‐understood. Additional findings revealed that African men presented with prostate cancer at higher rates than East Indian or mixed men in both the acute inpatient and elective outpatient settings. Although the general population of Trinidad and Tobago is 34% African and 35% East Indian according to the most recent census, 61% of the patients in this study identified as African and only 28% identified as East Indian.^11^ Since the study's survey method included all patients presenting to SFGH for prostate cancer care, sampling bias is less of a concern. However, it is possible that cultural discrepancies in symptom awareness or utilization of the public hospital system may be confounding variables that influenced our results. Surveys in private hospitals can help to better elucidate possible ethnic discrepancies.

Additionally, our analysis found that 20% of African patients had metastatic disease, while 14% of East Indian patients had metastatic disease. This pattern is consistent with other bodies of research evidencing that there is a racial disparity among prostate cancer patients in Trinidad. Previous studies showed not only higher rates of prostate cancer presentation in African men but also higher Gleason scores, PSA values and increased risk of mortality.^5^ At SFGH specifically, prostate cancer was three times as common in men of African descent compared to those of East Indian descent. These higher rates of presentation and metastatic disease correlate to higher costs for these patients. In this study, the average cost per African patient was 37% higher than the average cost per East Indian patient. Mixed patients also incurred a 14% higher cost than their East Indian counterparts. It is important to comment on these racial disparities to emphasize the need to address the social, economic and cultural barriers that may account for these demographic discrepancies and the far‐reaching effects of prostate cancer across the Trinidadian healthcare system.

Data that were not collected during this study included direct non‐medical costs, such as patient transportation to the hospital, and non‐direct costs, such as productive loss due to sick leave or premature disability and mortality due to prostate cancer. Because prostate cancer largely affects older men who may not be working (mean age of 69 years), indirect costs for patients can be assumed to be near zero. However, family members who frequently accompany patients to medical visits and are part of the working class may accrue significant indirect costs. Additional studies would need to be conducted to estimate the true financial burden on Trinidad's entire society, including patients and their families. Because other large public hospitals and various private facilities also provide urological care, it is difficult to estimate the entire cost associated with prostate cancer.

Another issue regarding the true cost of care relates to the concerted efforts made by SFGH to increase community‐based prostate cancer screening in the time during and after this study. While the decision to push community screening was unrelated to this investigation, it is important to note that costs may have been exaggerated during this study and thereafter because of the growing population identified for diagnosis and treatment. It is likely that not long ago in Trinidad, many men had been unknowingly suffering from prostate cancer sequelae that went unidentified until much later in their disease course when they presented with acute needs. Now, as a result of these screening efforts, the prevalence and cost of prostate cancer care at earlier stages and for diagnosis may account for a larger portion of costs.

Additional limitations of this study include potential missed cases due to the data collection process. Data were collected through in‐person surveys and paper chart reviews. Due to variable clinic schedules and limited availability of reporters to collect data across the hospital, some cases may have been missed. The nature of urology consult services across hospital specialties also leads us to believe that potential missed cases may have been more likely in the acute and inpatient setting. This is notable particularly because these acute presentations were consistently more costly. Additionally, some cases lacked a documented complaint for their visit, which may have led to variable findings comparing the costs of diagnosis versus follow‐up.

This study is, to our knowledge, the first to attempt to elucidate the financial burden of prostate cancer in the country of Trinidad and Tobago. Although the survey is reflective of only a single public hospital and is therefore not entirely comprehensive of Trinidad's public healthcare system, it estimates a significant portion of the cost to the Trinidadian government and patients. Because the study simultaneously examined multiple other parameters of cost and demographics, such as type of presentation/visit, ethnicity and metastatic disease, government budgets can be adjusted to initiate cost‐saving measures in certain departments, evaluate disease prevalence and continue to help populations most affected by prostate cancer.

## 5 CONCLUSION

The findings of this study highlight the public health importance of prostate cancer and the significant economic burden it places on the Trinidadian health system. Although prostate cancer investigations contribute to a large percentage of total costs, these earlier investigations provide enormous benefits in minimizing costs associated with more advanced treatment or acute hospitalization associated with later‐stage diagnosis and management. Patients with metastatic disease contributed disproportionately to overall costs of prostate cancer care, which is consistent with findings in other countries.^9^ African patients presented at a higher incidence and with higher rates of metastatic disease, leading to higher average costs per patient when compared to other ethnic Trinidadian counterparts. Efforts and funding toward prevention, screening and timely treatment of prostate cancer should be continued, as it will likely help patients avoid further disease progression and will lower the economic burden on Trinidad's healthcare system. Policymakers should continue to recognize the economic impact of the disease when making budgetary decisions and should consider initiating cost‐effective measures focused on early diagnosis when able to do so.

## AUTHOR CONTRIBUTIONS

Both Amanda Wibben and Geneva Pantoja have substantially and equally contributed to the authorship of this paper. Both authors performed the data collection and analysis and drafted the work. Satyendra Persaud contributed to the conception and design of the work, the acquisition and interpretation of the data, and the correspondence with officials at SFGH and T&T Ministry of Health. Sara Seetaram and Visham Bhagaloo contributed by identifying patient presentations across departments at SFGH and collecting data. Both Amanda Wibben and Geneva Pantoja critically reviewed the work for intellectual content, and both gave final approval of the version to be published. Both authors agree to be accountable for all aspects of the work.

## CONFLICT OF INTEREST STATEMENT

Funding for this research was provided by the Urology Cares Foundation International Student Humanitarian Summer Grant Program during the summer of 2023. Payments were made directly to Geneva Pantoja and Amanda Wibben to support travel and living expenses during the period of the study in Trinidad. The authors of this paper deny any related royalties, licences, events, payments, leadership roles, stock or other financial or non‐financial interests.

## References

[1] Sung H , Ferlay J , Siegel RL , Laversanne M , Soerjomataram I , Jemal A , et al. Global cancer statistics 2020: GLOBOCAN estimates of incidence and mortality worldwide for 36 cancers in 185 countries. CA Cancer J Clin. 2021;71(3):209–249. 10.3322/caac.21660 33538338

[2] Culp MB , Soerjomataram I , Efstathiou JA , Bray F , Jemal A . Recent global patterns in prostate cancer incidence and mortality rates. Eur Urol. 2020;77(1):38–52. 31493960 10.1016/j.eururo.2019.08.005

[3] Prostate cancer factsheet: globocan 2022 International Agency for Research on Cancer . World Health Organization . 2022

[4] Patrick A . Prostate cancer screening in an afro‐Caribbean population: the Tobago prostate cancer screening study. BJU Int. 2010;105(6):745–746. 10.1111/j.1464-410X.2010.09222.x 20353535

[5] Persaud S , Persaud M , Goetz L , Narinesingh D . The current state of prostate cancer treatment in Trinidad and Tobago. Ecancermedicalscience. 2018;12:828. 29743948 10.3332/ecancer.2018.828 PMC5931809

[6] Bunker CH , Patrick AL , Maharaj G , Keenan HA , Ramnarine S , Belle A , et al. Prostate cancer risk is three‐fold higher among men, aged 50–64, of African descent compared with men of Asian‐Indian descent in Trinidad and Tobago. Ethn Dis. 2002;12(4):S3. 12477151

[7] Zeigler‐Johnson CM , Spangler E , Jalloh M , Gueye SM , Rennert H , Rebbeck TR . Genetic susceptibility to prostate cancer in men of African descent: implications for global disparities in incidence and outcomes. Can J Urol. 2008;15(1):3872–3882. 18304397 PMC3064717

[8] King‐Okoye M , Arber A , Faithfull S . Beliefs that contribute to delays in diagnosis of prostate cancer among Afro‐Caribbean men in Trinidad and Tobago. Psychooncology. 2019 Jun;28(6):1321–1327. 10.1002/pon.5085 30953381 PMC6617795

[9] Mojahedian MM , Toroski M , Keshavarz K , Aghili M , Zeyghami S , Nikfar S . Estimating the cost of illness of prostate cancer in Iran. Clin Ther. 2019;1:50–58. 10.1016/j.clinthera.2018.11.001 30545740

[10] Mattiuzzi C , Lippi G . Current cancer epidemiology. J Epidemiol Glob Health. 2019;9(4):217–222. 10.2991/jegh.k.191008.001 31854162 PMC7310786

[11] Trinidad and Tobago 2011 Population and Housing Census: Demographic Report [Internet]. Central Statistics Office, 2011 [cited 2024 Apr 01]. Available from: https://cso.gov.tt/wp-content/uploads/2020/01/2011-Demographic-Report.pdf

